# Visceral Fat Area Measured by Abdominal Bioelectrical Impedance Analysis in School-Aged Japanese Children

**DOI:** 10.3390/jcm11144148

**Published:** 2022-07-17

**Authors:** Yuriko Abe, Ryousuke Tonouchi, Mitsuhiko Hara, Tomoo Okada, Eric H. Jego, Tetsuya Taniguchi, Tsugumichi Koshinaga, Ichiro Morioka

**Affiliations:** 1 Division of Medical Education, Nihon University School of Medicine, Tokyo 173-8610, Japan; abe.yuriko@nihon-u.ac.jp; 2 Medical Education Center, Nihon University School of Medicine, Tokyo 173-8610, Japan; jego.erichajime@nihon-u.ac.jp; 3 Department of Pediatrics and Child Health, Nihon University School of Medicine, Tokyo 173-8610, Japan; tono_macintosh@yahoo.co.jp (R.T.); m-hara@wayo.ac.jp (M.H.); tomokada@me.com (T.O.); 4 Department of Health and Nutrition, Faculty of Human Ecology, Wayo Women’s University, Ichikawa 272-8533, Japan; 5 Department of Liberal Arts, Nihon University School of Medicine, Tokyo 173-8610, Japan; taniguchi.tetsuya@nihon-u.ac.jp; 6 Department of Pediatric Surgery, Nihon University School of Medicine, Tokyo 173-8610, Japan; koshinaga.tsugumichi@nihon-u.ac.jp

**Keywords:** adiponectin, computed tomography, leptin, non-alcoholic fatty liver disease, waist circumference

## Abstract

Abdominal bioelectrical impedance analysis (aBIA) has been in use to measure visceral fat area (VFA) in adults. Accurately measuring visceral fat using aBIA in children is challenging. Forty-six school-aged Japanese children aged 6–17 years (25 boys and 21 girls) were included in this study. All were measured, and their VFA obtained using aBIA (VFA-aBIA) and abdominal computed tomography (CT) (VFA-CT) were compared. VFA-aBIA was corrected using the Passing–Bablok method (corrected VFA-aBIA). The relationships between corrected VFA-aBIA and obesity-related clinical factors were analyzed, including non-alcoholic fatty liver disease (NAFLD) and serum leptin and adiponectin levels. Boys had higher VFA-CT than girls (*p* = 0.042), although no significant differences were found in their waist circumference, waist-to-height ratio, and body mass index. The corrected VFA-aBIA using y = 9.600 + 0.3825x (boys) and y = 7.607 + 0.3661x (girls) correlated with VFA-CT in both boys and girls. The corrected VFA-aBIA in patients with NAFLD was higher than that in those without NAFLD. Serum leptin and adiponectin levels were positively and negatively correlated with corrected VFA-aBIA, respectively. In conclusion, corrected VFA-aBIA was clearly correlated with VFA-CT and was related to NAFLD and serum leptin and adiponectin levels in school-aged Japanese children.

## 1. Introduction

The relationship between abdominal obesity, especially visceral fat accumulation, and metabolic syndrome (MetS), fatty liver disease, type 2 diabetes mellitus, and cardiovascular disease risks is well-known [1,2]. Even in school-aged children, visceral fat accumulation is associated with a higher risk of metabolic and cardiovascular diseases [3,4,5]. There are several methods for measuring visceral fat area (VFA) [6]. The method is selected according to safety, economy, and required time. However, it has not been determined which is the best method for children, considering the advantages and limitations. To elaborate further, only abdominal computed tomography (CT) and magnetic resonance imaging (MRI) can directly measure VFA [6]. VFA can be measured accurately using abdominal CT in a short time [7,8,9]. However, this method carries risks associated with radiation. Although MRI can measure VFA without radiation, this method is costly, and the long examination time is a burden on children. Therefore, waist circumference (WC) is commonly used as a proxy for abdominal adipose tissue measurement [2]. The International Diabetes Federation and the National Heart, Lung, and Blood Institute have coordinated their MetS criteria recommending that WC is used as a simple index to measure abdominal fat [10]. However, WC does not distinguish between visceral fat and subcutaneous fat [11], despite these two types of fat accumulation having different risks [4].

To overcome this limitation, abdominal bioelectrical impedance analysis (aBIA) is an established method that is more accurate for measuring VFA in adults [12,13]. For aBIA, EW-FA90 (Panasonic Corporation, Osaka, Japan) was used, which is an authorized medical device in Japan (No. 22500BZX00522000). EW-FA90 estimated VFA using a formula that includes impedance (the voltage generated by the current flow between the umbilicus and back), WC, and sex [14]. Importantly, it is also useful because of its low cost and ease of use in adults. However, aBIA is not adapted for children because the body composition of children differs from that of adults [14]. EW-FA90 is the most popular aBIA machine in Japan [12], and may have the potential to assess individual health risks in children. The aim of this novel study was to measure VFA in school-aged Japanese children using EW-FA90 and abdominal CT, and to compare the obtained values. Moreover, we examined the relationship between VFA and obesity-related clinical factors, such as the presence of non-alcoholic fatty liver disease (NAFLD) and serum leptin and adiponectin levels.

## 2. Materials and Methods

### 2.1. Study Design and Subjects

Forty-eight school-aged children aged 6–17 years who visited outpatient clinics or were hospitalized at Nihon University Itabashi Hospital between 2018 and 2019 were recruited for this study. The selection criteria were designed to include patients who medically needed abdominal CT and exclude patients who had abdominal tumors, malformations in the abdomen, chromosomal abnormalities, or who could not stand on their own. Of the 48 children, 46 children underwent examination using EW-FA90. Of the 46 children, three obese children were diagnosed with type 2 diabetes mellitus and none drank alcohol. Among the 46 children, 38 underwent blood examination for serum leptin and adiponectin levels. Abdominal CT and laboratory examinations were performed at the discretion of the attending physician, depending on the patient′s condition.

The study protocol was approved by the Institutional Review Board of Nihon University Itabashi Hospital (RK-161213-05; date 17 March 2017). The procedures were in accordance with the ethical standards of the responsible committee on human studies and the Helsinki Declaration of 1975, as revised in 2008. All guardians provided written informed consent to allow their children to be included in the study.

### 2.2. Anthropometry and Laboratory Tests

Standing height and body weight were measured by experienced nurses. The body mass index (BMI) was calculated using the following formula: BMI = kg/m^2^. The BMI percentile was calculated based on the standard weights of sex-, age-, and height-matched participants using data from the Ministry of Education, Science, Sports, and Culture in Japan [15]. WC was measured at the level of the umbilicus in late exhalation while standing. Venous blood samples were collected to measure serum leptin and adiponectin levels using radio immunoassay and latex turbidimetric immunoassay, respectively [16,17].

### 2.3. Bioelectrical Impedance Analysis “EW-FA90” for Measurement of Abdominal Fat Area

The VFA measured by aBIA (VFA-aBIA) were measured using an aBIA machine EW-FA90 (Panasonic Corporation, Osaka, Japan), which is an authorized medical device in Japan (No. 22500BZX00522000). The cost of this main unit is JPY 900,000 and a measurement pad for one patient is JPY 3000. This aBIA machine is the most popular in Japan and is of a size where one person can carry it (https://b-healthy.jp.eww.panasonic.com/, accessed on 12 June 2022 (in Japanese)). The participants were measured using an EW-FA90 built-in belt that measured VFA and impedance while standing. It took a few minutes to measure VFA, and the participants did not experience pain. VFA and impedance of each subject were measured three times and the average was calculated. EW-FA90 estimated VFA using a formula that includes impedance (the voltage generated by the current flow between the umbilicus and back), WC, and sex. The voltage occurring at the flank to the flow of current between the umbilicus and back was significantly correlated with VFA. The constants of the formulas used in EW-FA90 have only been determined for adults [14]. The aBIA using EW-FA90 was performed by one examiner.

### 2.4. Abdominal CT Measurement of Visceral Fat Area and Subcutaneous Fat Area

The VFA measured by CT (VFA-CT) was determined using CT at the umbilical level. All participants underwent CT scanning in the supine position using an Aquilion CT scanner (Canon Medical Systems Corporation, Ohtawara, Japan). VFA was calculated using Fat Scan (East Japan Institute of Technology Co., Ltd., Hitachi, Japan), which is authorized medical software (No. 227AHBZX00035000). The software confirmed the accuracy of the visceral and subcutaneous fat area measurements [7]. The sum of the VFA and SFA was defined as the total fat area.

### 2.5. Definition of Obesity

The criteria for the diagnosis of obesity followed those of the American Academy of Pediatrics. According to the criterion, a BMI above the 95th percentile was defined as obesity [18].

### 2.6. Non-Alcoholic Fatty Liver Disease

According to the North American Society for Pediatric Gastroenterology, Hepatology, and Nutrition (NASPGHAN) clinical practice guidelines for the diagnosis and treatment of NAFLD in children, NAFLD is a diagnosis of exclusion requiring the presence of hepatic steatosis and exclusion of other causes of hepatic steatosis besides NAFLD. In addition, the NASPGHAN guidelines state that CT, although reasonably sensitive and specific for diagnosing hepatic steatosis, is not recommended for diagnostic purposes because of the risks associated with radiation [19]. In this study, the participants underwent abdominal CT for reasons other than diagnosing NAFLD. In addition, the attending physician excluded liver diseases other than fatty liver disease. Finally, experienced radiologists diagnosed NAFLD based on abdominal CT scans according to the NASPGHAN guidelines.

### 2.7. Statistical Analyses

Setting 0.60 of Spearman’s rank-order correlation coefficient as an effect size, the required sample size is 19 for a significance level of 5% and power of 80%. Therefore, we decided to recruit at least 20 boys and 20 girls.

The Wilcoxon rank sum test was used for age, height, weight, WC, BMI, BMI percentile, impedance-aBIA, VFA-aBIA, corrected VFA-aBIA, and VFA-CT between boys and girls. The Fisher′s exact test was used to compare % of NAFLD between boys and girls.

Between VFA-aBIA or corrected VFA-aBIA and VFA-CT, Spearman’s rank-order correlation coefficients (*ρ*) were calculated. Corrected VFA-aBIA was obtained by the Passing–Bablok analysis [20]. Bland–Altman plot analyses were also performed. The corrected VFA-aBIA was compared in patients between with NAFLD and non-NAFLD by the Wilcoxon rank sum test. *ρ* values were calculated Between the corrected VFA-aBIA and serum leptin or adiponectin levels. A *p*-value < 0.05 indicated statistical significance.

Passing–Bablok analyses were performed using Stat Flex (ver. 7, Osaka, Japan). Wilcoxon rank sum tests were performed using R (version 3.6.1). The other analyses were conducted using SAS version 9.4 (SAS Institute Inc., Cary, NC, USA).

## 3. Results

### 3.1. Characteristics of Participants

**Figure 1:**
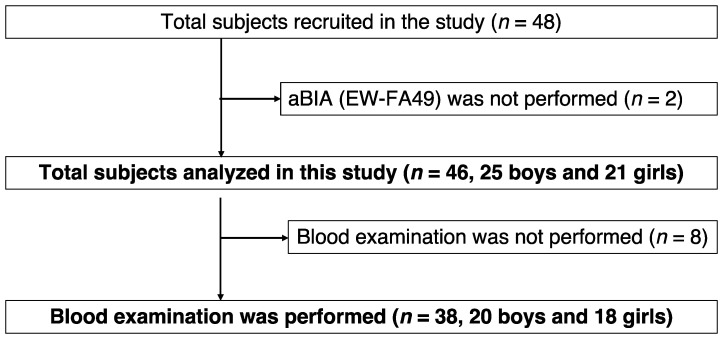
Flowchart of the study.

Among the 46 participants, 25 were boys and 21 were girls. Of the 46 participants, 38 underwent blood examination (Figure 1). The characteristics of the participants are shown in Table 1. There were no significant differences in age, height, body weight, WC, waist-to-height ratio, BMI percentile, or obesity rate between boys and girls. Both VFA-aBIA and VFA-CT were significantly higher in boys than in girls.

### 3.2. Relationship between VFA-aBIA or Corrected VFA-aBIA and VFA-CT

**Figure 2:**
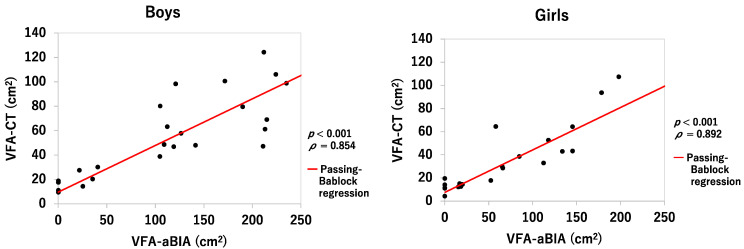
**Correlation between VFA-aBIA and VFA-CT in boys and girls.** VFA-aBIA—visceral fat area measured by abdominal bioelectrical impedance analysis using EW-FA90 (Panasonic Corporation, Osaka, Japan); VFA-CT—visceral fat area measured using computed tomography.

Figure 2 shows the relationship between the VFA-aBIA and VFA-CT. The numbers of VFA-aBIA were correlated with those of VFA-CT but were higher than those of VFA-CT. Therefore, because the body composition of children is different from that of adults, the VFA-aBIA values were corrected. For both boys and girls, the Passing–Bablok method assumed a linear relationship y = a + bx between VFA-aBIA and VFA-CT, y = 9.600 + 0.3825x (boys) and y = 7.607 + 0.3661x (girls). The value of y calculated by substituting the measured value (x’) of VFA-aBIA of each subject into x of the above linear relational expression was taken as the correlated VFA-aBIA.

**Figure 3:**
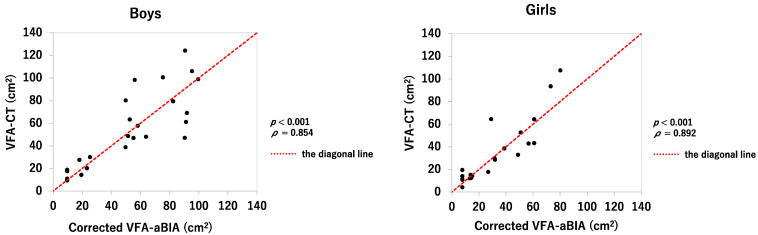
**Correlation between corrected VFA-aBIA and VFA-CT in boys and girls.** The corrected VFA-aBIA is VFA-aBIA corrected by the Passing–Bablok method: y = 9.600 + 0.3825x (boys) and y = 7.607 + 0.3661x (girls). VFA-aBIA—visceral fat area measured by abdominal bioelectrical impedance analysis using EW-FA90 (Panasonic Corporation, Osaka, Japan); VFA-CT—visceral fat area measured using computed tomography.

**Figure 4:**
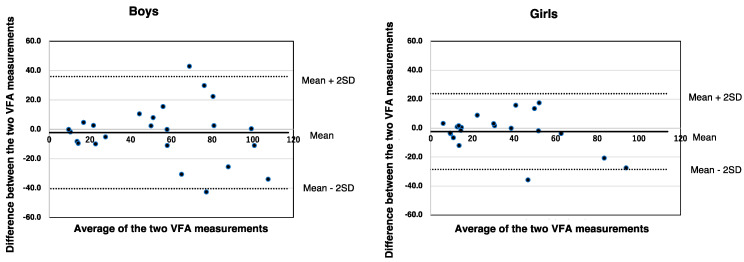
**The Bland–Altman plots for corrected VFA-aBIA and VFA-CT in boys and girls.** SD—standard deviation; VFA-aBIA—visceral fat area measured by abdominal bioelectrical impedance analysis using EW-FA90 (Panasonic Corporation, Osaka, Japan); VFA-CT—visceral fat area measured using computed tomography.

Corrected VFA-aBIA correlated significantly with VFA-CT in both boys and girls (*ρ* = 0.854, *p* < 0.001 and *ρ* = 0.892, *p* < 0.001, respectively; Figure 3). Figure 4 shows the Bland–Altman plot of VFA-aBIA and VFA-CT. The mean ± 2 standard deviations for corrected VFA-aBIA minus VFA-CT was −2.2 ± 38.2 (cm^2^) in boys and −2.4 ± 26.2 (cm^2^) in girls, respectively, indicating that the precision in girls was better than that in boys.

### 3.3. Relationship between VFA and Height, Body Weight, Waist Circumference, Waist-To-Height Ratio, or Body Mass Index

**Figure 5:**
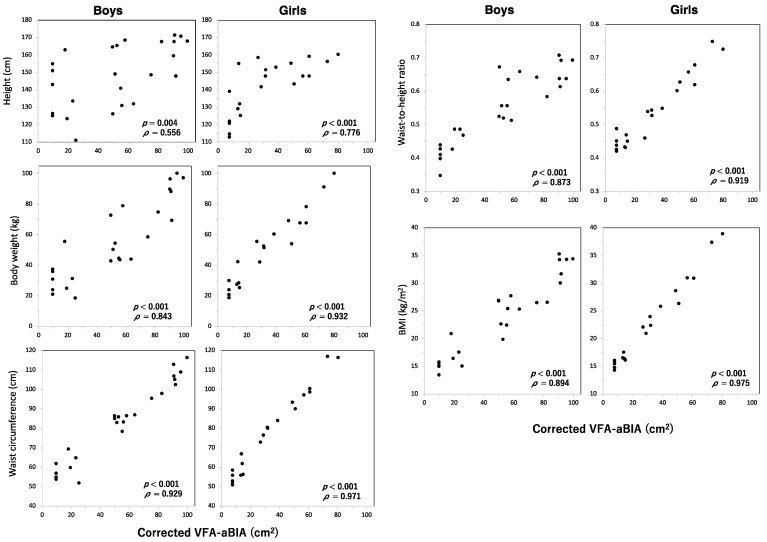
**Relationship between corrected VFA-aBIA and height, body weight, waist circumference, waist-to-height ratio, or body mass index.** VFA-aBIA—visceral fat area measured by abdominal bioelectrical impedance analysis using EW-FA90 (Panasonic Corporation, Osaka, Japan); BMI—body mass index.

Height, body weight, waist circumference, waist-to-height ratio, and BMI were positively correlated with corrected VFA-aBIA in boys and girls (*p* < 0.01, Figure 5).

### 3.4. Relationship between VFA and NAFLD

**Figure 6:**
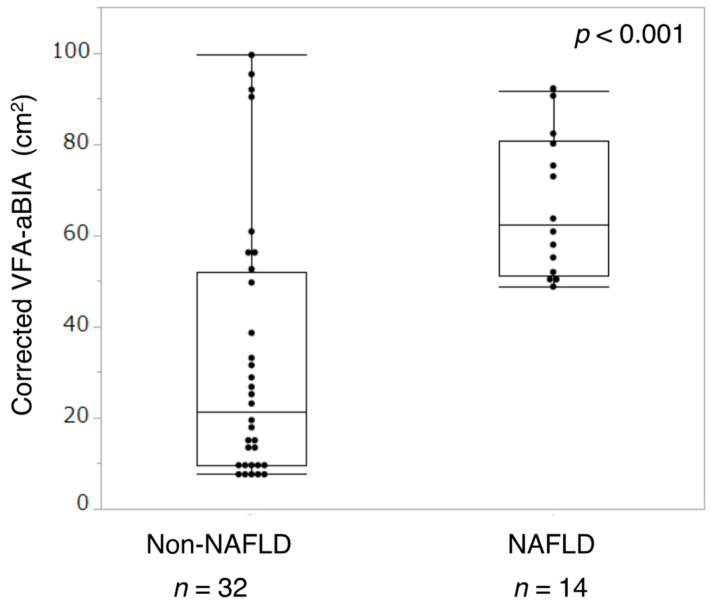
**Relationship between corrected VFA-aBIA and NAFLD.** VFA-aBIA—visceral fat area measured by abdominal bioelectrical impedance analysis using EW-FA90 (Panasonic Corporation, Osaka, Japan); NAFLD—non-alcoholic fatty liver disease.

Of all participants, 14 were diagnosed with NAFLD. The participants were divided into the NAFLD and non-NAFLD groups. The corrected VFA-aBIA in the NAFLD group was higher than that in the non-NAFLD group (*p* < 0.001, Figure 6).

### 3.5. Relationship between VFA and Serum Leptin and Adiponectin

**Figure 7:**
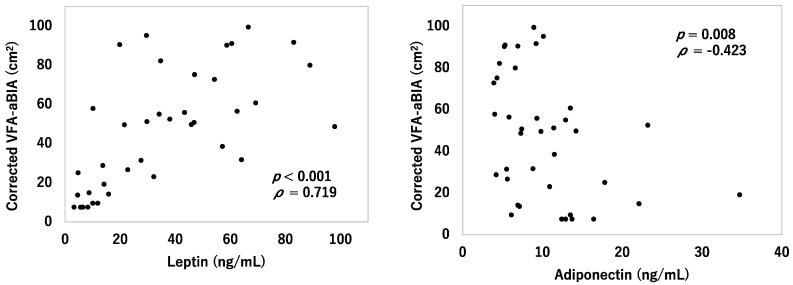
**Relationship between corrected VFA-aBIA and serum leptin or adiponectin levels.** VFA-aBIA—visceral fat area measured by abdominal bioelectrical impedance analysis using EW-FA90 (Panasonic Corporation, Osaka, Japan).

Figure 7 shows the relationship between corrected VFA-aBIA and serum leptin or adiponectin levels. Leptin levels were positively correlated with corrected VFA-aBIA (*ρ* = 0.719, *p* < 0.001). Adiponectin levels were negatively correlated with corrected VFA-aBIA (*ρ* = −0.423, *p* = 0.008).

## 4. Discussion

Obesity has become one of the leading causes of chronic metabolic diseases and cardiovascular disease, affecting not only adults but children as well [1]. Moreover, pediatric obesity often causes long-term health complications and threatens adult health [21]. Especially among the obese, a large VFA is a risk factor for type 2 diabetes mellitus [4]. Therefore, it is required to measure VFA safely and easily in children. Several methods of VFA measurement have been reported [6], but it has not been determined which method is best for children. Currently, only CT or MRI can accurately measure VFA. However, CT is associated with the problem of radiation exposure. MRI does not involve radiation but is costly and the facilities are limited. WC is a simple method but is affected not only by VFA but also by subcutaneous fat. Ultrasound and dual-energy X-ray absorptiometry have also been used, but data on children are scarce.

In adults, aBIA was used to measure the VFA. aBIA is a new method of quantifying VFA by combining information about impedance and abdominal shape, which can be measured using WC or abdominal dimensions using built-in calipers [6]. There are several types of aBIA machines, and the accuracy of VFA measurement has been reported in the past. For example, VFA measured using the aBIA machine EW-FA90, which we used in this study, correlated with VFA measured using CT [12]. EW-FA90 is the most popular aBIA machine in Japan and is used for adult outpatients and health examinations. In a study of participants aged 19 to 58 years, VFA measured using aBIA, ultrasound, and CT were compared. In participants with BMI lower than 25, BIA best correlated with VFA measured using CT. In contrast, in participants with a BMI greater than 30, the waist–hip ratio showed the best correlation with VFA measured using CT [22]. In another study using other aBIA machines, VFA measured using aBIA was compared with VFA performed from abdominal MRI and concluded that the aBIA machine is a useful measure of both abdominal fat and WC [23]. In addition, some studies suggest that aBIA tends to underestimate VFA when VFA is high [24,25]. In contrast, it is not known if VFA could be measured using aBIA in children.

The major advantages of aBIA are that it is a non-radiation imaging tool, low cost, easy to use, portable, and takes only a few minutes. These benefits are particularly beneficial in children. Therefore, in this study, we measured VFA using aBIA and compared VFA-aBIA to VFA-CT. First, we used EW-FA90 as a medical device in Japan. The aBIA machine calculates VFA from the formula with impedance, sex, and WC as parameters [12]. Impedance is affected by the amount of water, and total body water in children and adults is different [26]. Previously, aBIA for VFA measurement has not been indicated for children because the formula could not be applied to children. A recent report has shown that total body water in children can be accurately measured using aBIA [26]. From the background, it would be economical and easy if the machine already used by adults could be used for children by changing only the formula.

In the present study, we measured VFA using EW-FA90. As expected, VFA-aBIA values deviated significantly from those of VFA-CT. We showed for the first time that the value of VFA-aBIA was significantly higher than that of VFA-CT. In other words, an aBIA machine for adults cannot be directly adapted to children. Therefore, it is necessary to create a new formula for children, as hypothesized. From the obtained data, we created a pediatric formula to correct the VFA values. The corrected VFA-aBIA was correlated with VFA-CT. In addition, when VFA was large, aBIA tended to underestimate VFA, similarly to reports of adult participants. Another important factor is that VFA-aBIA correlates with metabolic risk factors such as NAFLD and adipocytokines. Therefore, aBIA is considered to be a viable method for managing metabolic diseases by combining physique measurements in school-aged children.

It has been reported that NAFLD in obese children is strongly associated with multiple MetS criteria [27]. NAFLD is initially asymptomatic and may be delayed in detection. However, because NAFLD may progress to serious liver disease, early detection is desirable. In our study, participants diagnosed with NAFLD using CT had higher VFA-aBIA than those without NAFLD. Usually, abdominal echo, CT, and MRI are used to diagnose NAFLD, but all require expensive machines and skilled techniques. From our results, it was found that aBIA could be used to easily predict the risk of NAFLD before diagnosis using special machines.

Another finding of our study was that leptin and adiponectin levels were correlated with VFA-aBIA. Leptin is potentially implicated in obesity-related cardiovascular disease [28]. Because potentially harmful effects of leptin may be countered by other pleiotropic effects, it has also been reported that leptin levels are not associated with incidences of cardiovascular events in adults in clinical settings [29,30]. In contrast, blood adiponectin concentrations decreased in obese patients, and decreased adiponectin leads to obesity-related disorders such as type 2 diabetes mellitus and cardiovascular disease [29,31]. It has been reported that low blood adiponectin concentration independently predicted future abdominal visceral fat accumulation and increased insulin resistance in adults [32]. In addition, the relationship between VFA and leptin and adiponectin levels has not been clarified in children. In the present study, leptin levels positively correlated with VFA-aBIA. Conversely, adiponectin levels negatively correlated with VFA-aBIA levels. This suggests that adipocytokines are associated with VFA.

In the present study, the participants were divided into boys and girls because the aBIA machine we used calculated VFA separately for men and women. There were no differences in age, height, weight, WC, waist-to-height ratio, BMI, BMI percentile, or obesity rates. Surprisingly, boys had a higher VFA-CT than girls, even though there was no significant difference in physique between boys and girls. In adults, it was reported that men have a higher tendency to accumulate VFA compared to pre-menopausal women [33]. In addition, it was reported that the Asian population is more susceptible to metabolic derangement induced by mild obesity than the Caucasian population [34]. There are few reports about the difference in VFA in children. In this study, Japanese boys were considered more likely to accumulate VFA than Japanese girls.

The major limitation of this study was the small number of participants. However, the physique of the participants did not differ between boys and girls and included obesity and non-obesity. Therefore, this study included a wide range of participants. Our findings are valuable given the paucity of investigations of VFA measured using aBIA in school-aged children.

## 5. Conclusions

This is the first study to demonstrate that EW-FA90 is a useful measurement tool for school-aged children by using the calculation formula for children. The corrected VFA measured using EW-FA90 was correlated with VFA-CT, NAFLD, and serum leptin and adiponectin levels. By converting this machine for pediatric use, EW-FA90 can be safe and easy to use repeatedly for prediction metabolic risks in school-aged children and determination of the treatment effectiveness in school-aged obese children.

## Figures and Tables

**Table 1:** Characteristics of boys and girls.

	Boys, *n* = 25	Girls, *n* = 21	*p*-Value
Age (year)	12 (6–17)	10 (7–17)	0.632
Height (cm)	149.1 (111.0–171.4)	147.9 (112.9–160.4)	0.142
Body weight (kg)	50.5 (18.6–100.2)	51.6 (18.9–100.3)	0.372
Waist circumference (cm)	85 (52.0–116.5)	76.5 (51.0–117.0)	0.384
Waist-to-height ratio	0.56 (0.35–0.71)	0.53 (0.42–0.75)	0.662
BMI (kg/m^2^)	25.4 (13.5–35.3)	22.1 (14.4–39.0)	0.674
BMI percentile (%)	95.1 (4.2–99.9)	90.0 (17.6–99.9)	0.861
Impedance-aBIA (Ω)	1495 (619–2969)	1586 (645–2140)	0.972
VFA-aBIA (cm^2^)	112.3 (0.0–235.0)	58.0 (0.0–198.0)	0.110
Corrected VFA-aBIA (cm^2^)	52.6 (9.6–99.5)	28.8 (7.6–80.1)	0.035 *
VFA-CT (cm^2^)	48.1 (9.6–124.4)	28.7 (4.3–107.6)	0.042 *
NAFLD (%)	36	24	0.522

Data are presented as medians (ranges) or percentages. The Fisher′s exact test was used for % NAFLD, and the Wilcoxon rank-sum test was used for others. aBIA—abdominal bioelectrical impedance analysis; BMI—body mass index; CT—computed tomography; NAFLD—non-alcoholic fatty liver disease; SFA—subcutaneous fat area; VFA—visceral fat area. * *p*-values indicate statistical significance.

## Data Availability

The data that support the findings of this study are available from the corresponding author upon reasonable request.
